# Possible molecular basis of the biochemical effects of cysteine-derived persulfides

**DOI:** 10.3389/fmolb.2022.975988

**Published:** 2022-09-23

**Authors:** Ernesto Cuevasanta, Dayana Benchoam, Jonathan A. Semelak, Matías N. Möller, Ari Zeida, Madia Trujillo, Beatriz Alvarez, Darío A. Estrin

**Affiliations:** ^1^ Laboratorio de Enzimología, Instituto de Química Biológica, Facultad de Ciencias, Universidad de la República, Montevideo, Uruguay; ^2^ Unidad de Bioquímica Analítica, Centro de Investigaciones Nucleares, Facultad de Ciencias, Universidad de la República, Montevideo, Uruguay; ^3^ Centro de Investigaciones Biomédicas (CEINBIO), Universidad de la República, Montevideo, Uruguay; ^4^ Graduate Program in Chemistry, Facultad de Química, Universidad de la República, Montevideo, Uruguay; ^5^ Departamento de Química Inorgánica, Analítica y Química Física, Instituto de Química Física de los Materiales, Medio Ambiente y Energía (INQUIMAE), Facultad de Ciencias Exactas y Naturales, Universidad de Buenos Aires and CONICET, Buenos Aires, Argentina; ^6^ Laboratorio de Fisicoquímica Biológica, Instituto de Química Biológica, Facultad de Ciencias, Universidad de la República, Montevideo, Uruguay; ^7^ Departamento de Bioquímica, Facultad de Medicina, Universidad de la República, Montevideo, Uruguay

**Keywords:** cysteine, persulfide, alpha effect, hydrogen sulfide, sulfhydryl

## Abstract

Persulfides (RSSH/RSS^−^) are species closely related to thiols (RSH/RS^−^) and hydrogen sulfide (H_2_S/HS^−^), and can be formed in biological systems in both low and high molecular weight cysteine-containing compounds. They are key intermediates in catabolic and biosynthetic processes, and have been proposed to participate in the transduction of hydrogen sulfide effects. Persulfides are acidic, more acidic than thiols, and the persulfide anions are expected to be the predominant species at neutral pH. The persulfide anion has high nucleophilicity, due in part to the alpha effect, i.e., the increased reactivity of a nucleophile when the neighboring atom has high electron density. In addition, persulfides have electrophilic character, a property that is absent in both thiols and hydrogen sulfide. In this article, the biochemistry of persulfides is described, and the possible ways in which the formation of a persulfide could impact on the properties of the biomolecule involved are discussed.

## Introduction

Persulfides (RSSH/RSS^−^)^1^, close relatives of thiols (RSH/RS^−^) and hydrogen sulfide (H_2_S/HS^−^), are sulfane sulfur compounds, i.e., compounds that contain sulfur bound to two sulfur atoms or to a sulfur and an ionizable hydrogen. They can be formed in biological systems through processes that are dependent or independent of H_2_S/HS^−^, and they have prominent roles in sulfur trafficking and metabolism. They are intermediates in the mitochondrial oxidation of H_2_S/HS^−^ (Hildebrandt and Grieshaber, 2008) and in the biosynthesis of thionucleosides, iron-sulfur clusters and other cofactors (Mueller, 2006). Persulfides are receiving increased attention as possible intermediates in H_2_S/HS^−^ signaling. A few decades ago, the classical view that H_2_S/HS^−^ was simply a poisonous gas started to evolve when it was acknowledged that H_2_S/HS^−^ can be synthesized and catabolized in mammalian tissues and that, at low concentrations, it can exert physiological effects with potential health benefits, particularly in the cardiovascular and nervous systems (Abe and Kimura, 1996; Hosoki et al., 1997). The formation of persulfides downstream of H_2_S/HS^−^ could be at the basis of at least some of the effects ascribed to it.

**FIGURE 1:**
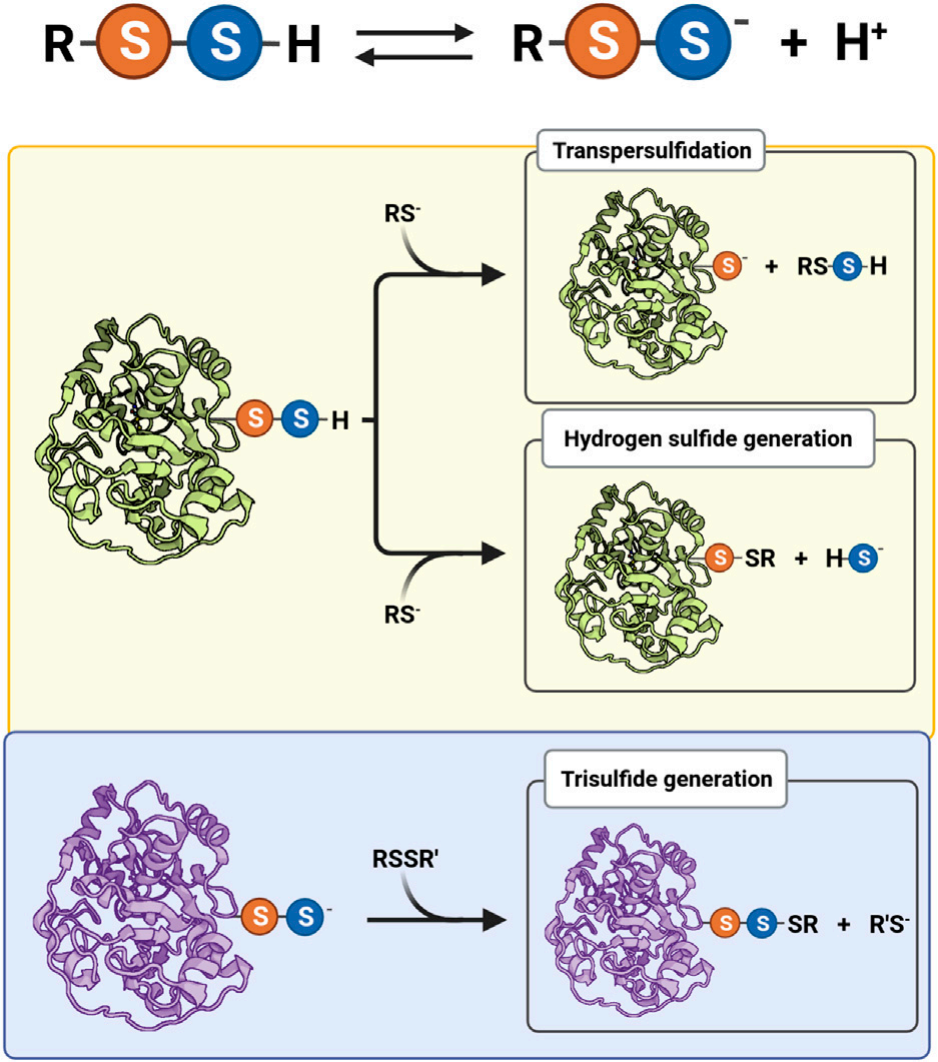
Acid-base equilibrium and reactivity of biological hydropersulfides. Protonated (RSSH) and ionized persulfides (RSS^−^) are in equilibrium, affecting their reactivity. The electrophilic behavior of protonated persulfides (yellow background) is exemplified by a protein persulfide and a thiolate (RS^−^) as nucleophile. The nucleophilic behavior of persulfides (light blue background) is illustrated by a protein persulfide and a generic disulfide (RSSR′) as electrophile.

Persulfides tend to be more acidic and better nucleophiles than thiols and H_2_S/HS^−^ because the anions are more available at physiological pH and have increased intrinsic reactivity due to the alpha effect, i.e., the increased reactivity of a nucleophile when the neighboring atom has high electron density (Cuevasanta et al., 2015; Benchoam et al., 2020). Besides, persulfides have an additional property that is absent in thiols and in H_2_S/HS^−^: electrophilicity (Figure 1). Not surprisingly, persulfides are unstable in aqueous solution, which challenges the study of their properties.

In this minireview, we briefly summarize the biochemistry of persulfides and discuss the possible ways in which the formation of a persulfide could impact on the properties of the parent cysteine-containing biomolecule, providing examples.

### Formation of persulfides in biological systems

Several pathways that lead to the formation of persulfides involve H_2_S/HS^−^, thiols and oxidants. Some of the reactions implicated show correspondence with well-known redox reactions observed for thiols. Oxidized thiol derivatives, like sulfenic acids, disulfides and trisulfides, are prone to be attacked by ${HS}^{-}$ , the conjugate base of H_2_S (Eqs. 1–3). The attack on the sulfur leads to a persulfide and to secondary leaving products (hydroxide, thiolate and persulfide, respectively).

$$RSOH+HS^{-}\to RSSH+{OH}^{-}$$ (1)

$${RSSR}^{\prime }+HS^{-}⇌RSSH+R^{\prime }S^{-}$$ (2)

$${RSSSR}^{\prime }+HS^{-}⇌RSSH+R^{\prime }{SS}^{-}$$ (3)

The reaction that involves sulfenic acids (Eq. 1) was characterized in model proteins like human serum albumin and alkyl hydroperoxide reductase E from *Mycobacterium tuberculosis* (*Mt*AhpE, a one-cysteine peroxiredoxin, particularly fast-reacting with hydroperoxides) (Cuevasanta et al., 2015, 2019). These proteins form sulfenic acids that are transiently stabilized by the protein environment. The reaction between sulfenic acids and HS^−^ explains the marked increases in persulfide levels observed in cell cultures treated with H_2_O_2_ and H_2_S/HS^−^ (Cuevasanta et al., 2015).

The attack of HS^−^ on a disulfide (Eq. 2) is analogous to the thiol-disulfide exchange reaction (Cuevasanta et al., 2015). This process occurs, for example, in the active site of flavocytochrome *c* sulfide dehydrogenases, which is the first step in the catalysis performed by this sulfide-oxidizing prokaryotic enzyme (Tikhonova et al., 2021). A similar reaction is observed in the catalytic site of the human sulfide quinone oxidoreductase (SQOR), the enzyme responsible for mitochondrial H_2_S/HS^−^ oxidation. The electrophile, in this case, is an internal trisulfide (Eq. 3) (Landry et al., 2019). Although the reaction of HS^−^ with disulfides is relatively slow in solution, the protein environment and the presence of a better leaving group in trisulfides (a second persulfide, Eq. 3) can facilitate catalysis.

Alternatively, persulfides can be formed from the reaction of reduced thiols with oxidized H_2_S/HS^−^ derivatives. Organic and inorganic polysulfides ( ${RSS}_{n}H,{HSS}_{n}H,n>1$ ) as well as thiosulfate ( $SSO_{3}^{2-}$ ) are generated from H_2_S/HS^−^ oxidation. Thiolates can accept the outer sulfurs to form persulfides (Eq. 4).

$${RS}^{-}+{HSS}_{n}H⇌RSSH{+}^{-}S_{n}H$$ (4)

Enzymes like thiosulfate sulfurtransferase (rhodanese) are able to catalyze the transfer of the sulfur from thiosulfate or low molecular weight (LMW) persulfides to varied acceptors, including thiols (Cipollone et al., 2007).

Finally, other pathways that do not involve H_2_S/HS^−^ require particular enzymatic systems. Cystathionine β-synthase and cystathionine γ-lyase, which are PLP-containing enzymes, can use cystine to form the LMW cysteine persulfide through α,β-elimination (Yadav et al., 2016). Other enzymes are able to form a persulfide in an active site cysteine that is then transferred to fulfill either catabolic or biosynthetic roles. The PLP-dependent cysteine desulfurases take the sulfur of free cysteine to form an internal persulfide. The sulfane sulfur is transferred to other proteins for synthesizing iron-sulfur clusters, molybdopterin and thionucleosides, among others (Kim and Park, 2013). 3-Mercaptopyruvate sulfurtransferases (MPSTs), however, obtain sulfur from 3-mercaptopyruvate with no assistance of cofactors (Pedre and Dick, 2021). They are usually involved in catabolic pathways and transfer the sulfur to thiophilic acceptors, like thiols (to form persulfides or H_2_S/HS^−^) or cyanide (to form thiocyanate).

### Properties of persulfides

The RSSH species is more acidic than its thiol analog, at least in LMW compounds (Figure 1). This fact is mainly due to a weaker S-H bond in the case of RSSH. For example, the persulfide of glutathione (GSSH) has a p*K*_a_ of 5.5, while the p*K*_a_ of glutathione (GSH) is 3.5 units higher (Everett et al., 1994; Portillo-Ledesma et al., 2014; Benchoam et al., 2020). Both persulfides and thiols react as nucleophiles mainly in the RSS^−^ and RS^−^ forms. The negative charge in RSS^−^ species is mostly located on the outer nucleophilic sulfur atom. The persulfide anion is expected to react faster than a thiolate of equal basicity, according to the alpha effect (Fina and Edwards, 1973). Additionally, depending on the electrophile, the persulfide anion can also be more nucleophilic than its thiolate structural analog (Benchoam et al., 2020). Importantly, it should be mentioned that the reactions of RSS^−^ acting as nucleophiles form products that contain two electrophilic sulfur atoms (Figure 1), which make it possible to recover the thiol species by a subsequent nucleophilic attack to the original outer sulfur.

The observed reactivity of persulfides at a certain pH responds to the combination of the intrinsic reactivity of the RSS^−^ species and its availability. In the case of glutathione persulfide, GSS^−^ availability is 99% at pH 7.4. In contrast, the availability of GS^−^ is only 2.8% at the same pH. The differences in reactive species availability decrease in cellular compartments with a more alkaline pH such as in the mitochondrial matrix (pH 7.8–8). Besides, the intrinsic reactivity of GSS^−^ in comparison with GS^−^ is increased 1.8-fold with peroxynitrite, and 44-fold with monobromobimane. Free energy profile simulations indicated that this difference is related to the electronic structures of the species rather than to solvent effects. The combination of higher intrinsic reactivity and increased availability results in GSSH reacting 97 and 1,200 times more rapidly than GSH with peroxynitrite and monobromobimane, respectively, at neutral pH. Additionally, the increased reactivity of GSS^−^ compared not with GS^−^ but with a thiolate of similar basicity is 50-fold with peroxynitrite and 1,670-fold with monobromobimane. Thus, the magnitude of the alpha effect varied with the electrophile. Indeed, this magnitude appeared to increase with the Brønsted β_nuc_ values of the corresponding reactions of LMW thiolates with each electrophile, which in turn correlate with the amount of charge transferred in the transition state (Benchoam et al., 2020).

**FIGURE 2:**
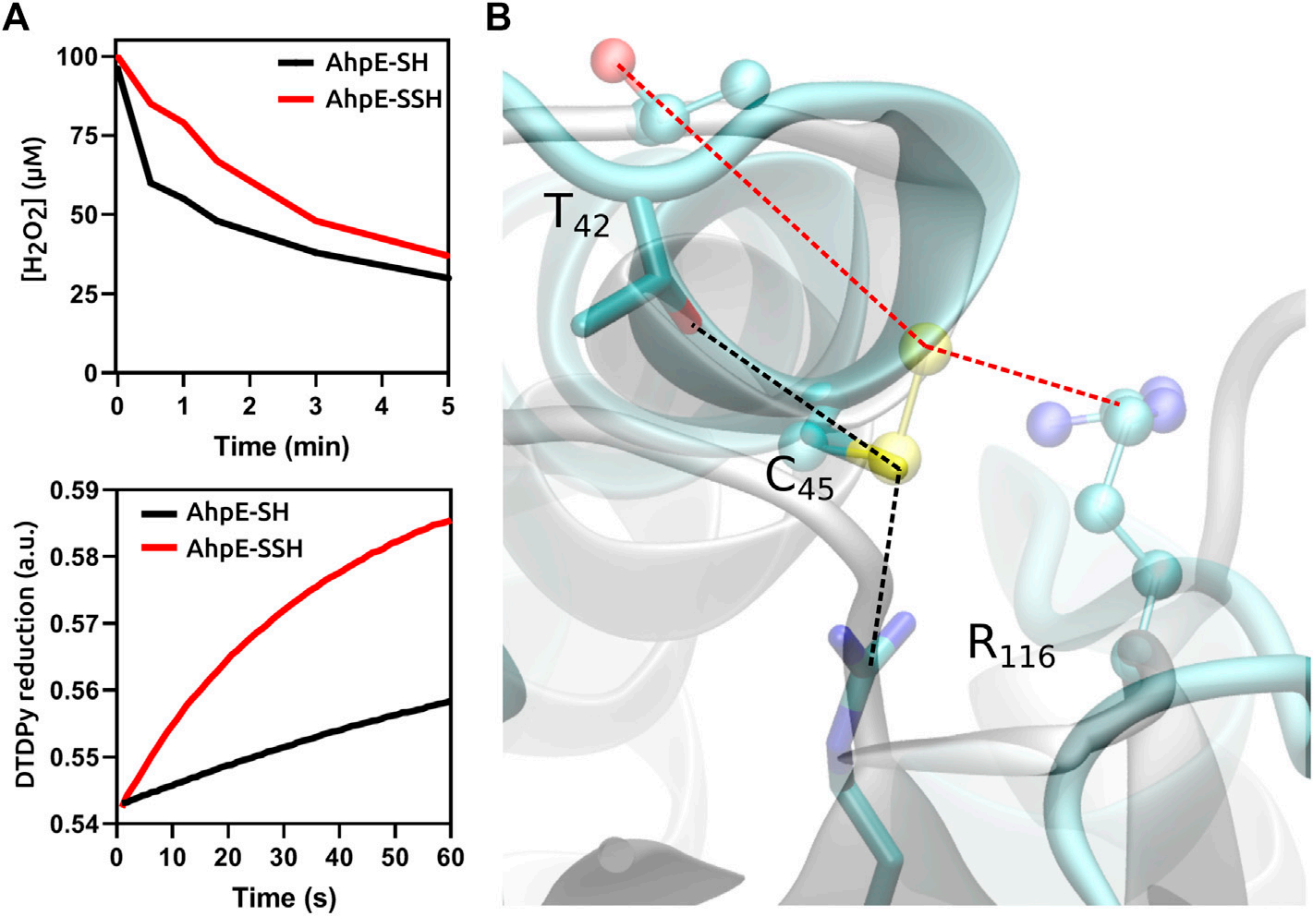
Functional and structural consequences of the persulfidation of the reactive cysteine in *Mt*AhpE. **(A)** Time courses comparison of the reactions of native (thiol, black) and persulfidated (red) enzymes with H_2_O_2_ (one of its physiological substrates, top) or 4,4′-dithiodipyridine (DTDPy, synthetic unspecific substrate, bottom) as electrophiles. **(B)** Superposition of representative structures of native (cyan, sticks) and persulfidated (gray, balls and sticks) *Mt*AhpE active sites. Dotted lines indicate the distances of the key interactions among the peroxidatic cysteine and threonine or arginine residues that form the catalytic triad (black, native; red, persulfidated). Modified from (Cuevasanta et al., 2019).

In the case of protein cysteines, the effect of persulfidation on the nucleophilic reactivity compared with the corresponding thiol is variable, and depends not only on the particular protein but also on the electrophile as well as on experimental conditions such as pH. Persulfidation of the single reduced cysteine residue of human serum albumin caused an increase in reactivity towards both peroxynitrite and the disulfide 4,4′-dithiodipyridine (Cuevasanta et al., 2015). In contrast, the persulfidation of the critical cysteine in the peroxiredoxin *Mt*AhpE caused an increase in the reactivity with 4,4′-dithiodipyridine, which is not a substrate for the enzyme, but inhibited the reactivity towards the physiological substrates H_2_O_2_ and peroxynitrite, when compared with the unmodified protein in the reduced state, due to alterations in the structure of the active site (Figure 2) (Cuevasanta et al., 2019). From these data it becomes clear that persulfidation effects in protein cysteine nucleophilic reactivity are dependent on the target electrophile and on the protein itself.

Besides being nucleophiles, persulfides are also electrophiles, a property that is absent in thiols and in H_2_S/HS^−^, but that is shared with polysulfides. Both sulfur atoms of persulfides (actually, protonated persulfides) are subject to nucleophilic attack (Figure 1). Different products are formed depending on the site of the attack, which in turn depends on the nature of the leaving group. When the inner sulfur is attacked, HS^−^ and a sulfur-bridged derivative are formed (Eq. 5). If the nucleophile is a thiolate, a mixed disulfide is the derivative produced (Eq. 6). For instance, the reaction of GSH and GSSH forms H_2_S/HS^−^ and GSSG (Benchoam et al., 2020).

$$RSSH+{Nu}^{-}\to RSNu+{HS}^{-}$$ (5)

$$RSSH+R^{\prime }S^{-}\to RS{SR}^{\prime }+{HS}^{-}$$ (6)

When the outer sulfur is attacked, the products are a thiolate and a sulfur derivative (Eq. 7). This reaction is particularly relevant when the nucleophile is a thiolate. In that case, a transpersulfidation reaction occurs and a new persulfide is formed at the attacking thiol (Eq. 8).

$$RSSH+{Nu}^{-}\to RS^{-}+NuSH$$ (7)

$$RSSH+R^{\prime }S^{-}\to RS^{-}+R^{\prime }SSH$$ (8)

Transpersulfidation reactions constitute the basis for the reaction mechanisms of several enzymes. For example, the catalytic mechanism of MPST involves the formation of the persulfidated protein and the subsequent transfer of the sulfane sulfur to a thiol acceptor, such as cysteine or thioredoxin (Figure 1) (Yadav et al., 2020; Pedre and Dick, 2021). Additional examples include other sulfurtransferases, such as rhodanese, that can react with LMW persulfides to generate the persulfidated protein (Melideo et al., 2014; Libiad et al., 2018). Furthermore, the persulfide formed in *Mt*AhpE was able to transfer the sulfane sulfur to a thiol-containing fluorogenic probe (Cuevasanta et al., 2019). The prokaryotic thiol-based transcriptional repressor SqrR takes advantage of persulfide electrophilicity to sense GSSH. After transpersulfidation and acquisition of several sulfur atoms, it forms an internal tetrasulfide which induces a decrease in the protein affinity for the target DNA sequence (Capdevila et al., 2021).

In addition to thiolates, other nucleophiles can attack the outer sulfur in persulfides. These include cyanide (Eq. 9), sulfite (Eq. 10), amines, hydroxide and tertiary phosphines (Filipovic et al., 2018).

$$RSSH+{CN}^{-}\to RS^{-}+HSCN$$ (9)

$$RSSH+SO_{3}^{2-}\to RS^{-}+{HSSO}_{3}^{-}$$ (10)

The enzyme SQOR contains a trisulfide in its active site that forms two persulfides when attacked by HS^−^ in the first reaction of H_2_S/HS^−^ mitochondrial detoxification (Landry et al., 2019). While the persulfide formed at Cys_201_ forms a charge transfer complex with the flavin cofactor, the persulfide at Cys_379_ has remarkable substrate promiscuity, and is susceptible to the attack of several acceptors *in vitro*. Some examples include thiols, such as GSH and coenzyme A (which form GSSH and coenzyme A persulfide, respectively), H_2_S/HS^−^, sulfite (to give thiosulfate) and cyanide (to give thiocyanate) (Landry et al., 2021).

Finally, considering that persulfides are both nucleophilic and electrophilic, it would be reasonable to think that they may react with each other. Indeed, persulfides decay spontaneously, ultimately forming elemental sulfur, polysulfides, H_2_S/HS^−^, thiols and disulfides.

### Physiological implications of cysteine persulfidation

The formation of persulfides in cysteines may induce changes in the properties of the original thiol in different ways. Although detailed mechanistic studies are scarce, several examples of alteration of function after persulfide formation have been reported.

Different subtypes of K^+^ channels respond to H_2_S/HS^−^ levels. Among them, persulfidation of a single cysteine in the Kir 6.1 subunits of vascular ATP-sensitive K^+^ channel increases the activity, causing endothelial and smooth muscle cell hyperpolarization and vasorelaxation (Mustafa et al., 2011). It was proposed that persulfide formation introduces a negative charge in the electropositive ATP binding site. Recently, H_2_S/HS^−^ levels have been related to the severity of Covid-19 disease through the persulfidation and activation of ATP-sensitive channels of leukocytes, which is leading to novel therapeutic interventions (Dattilo, 2020). In other K^+^ channels, cysteine persulfidation causes inactivation.

A different mechanism acts in mammalian aquaporin 8, a transporter that allows the flux of H_2_O_2_ across cellular membranes. It was shown that gating is produced due to persulfidation of a cysteine close to the narrow channel after treatment with H_2_S/HS^−^, probably preceded by a transient sulfenic acid intermediate (Bestetti et al., 2018). Adding an extra sulfur atom to the cysteine distorts the constriction site, hindering transport through the channel.

Many cases have been reported in which the persulfidation of cysteines in active sites of enzymes disrupted atomic distances and hydrogen bonding tailored for catalysis. This effect was discussed above for the peroxiredoxin *Mt*AhpE (Figure 2). Similar inhibitory effects are expected to occur in other peroxiredoxins, since the active site is strongly conserved among different members of the protein family. Since peroxiredoxins are the main reductases for different hydroperoxides such as H_2_O_2_ and peroxynitrite, persulfidation would cause an increase in cellular steady-state levels of these oxidants. However, the situation is not so simple since the final effect will depend also on the rates of reduction of persulfidated versus hyperoxidized enzymes. Similarly, critical cysteine persulfidation causes protein tyrosine phosphatase 1B (PTP1B) (Krishnan et al., 2011) and glyceraldehyde-3-phosphate dehydrogenase (GAPDH) (Jarosz et al., 2015) inhibition, although there is debate in the literature related to the latter (Fu et al., 2020). The inhibition of those enzymes may impact the phosphorylation cascades and the intensity of the flux in glycolysis, respectively. Other enzymes like tyrosine aminotransferase and papain are also inhibited by incubation with LMW persulfides that are able to transfer the external sulfur to critical cysteines (Hargrove, 1988; Francoleon et al., 2011).

Other examples would comprise large structural changes. Persulfidation has been suggested to be involved in Keap1 regulation, where H_2_S/HS^−^ is postulated to react with an internal disulfide decreasing its affinity for Nrf-2 and leading to changes in gene expression (Hourihan et al., 2013). A similar mechanism was postulated for RAGE proteins. The formation of persulfides would impede the assembling of functional dimers of the receptor, leading to cell protection against cellular death and senescence (Zhou et al., 2017).

The formation of a sulfenic acid mediates the epidermal growth factor receptor (EGFR) activation enhancing its tyrosine kinase activity, and it has been suggested that the reaction of H_2_S/HS^−^ to form a persulfide could work as a switch to control the duration of the signaling (Zivanovic et al., 2019), by limiting the downstream effects of the growth factor. Also, an activation mechanism involving an attack of H_2_S/HS^−^ at a disulfide in the intracellular kinase core was postulated for EGFR regulation (Ge et al., 2014) that would promote Na^+^/K^+^-ATPase endocytosis in renal tubular epithelial cells through a downstream signaling pathway. Consequently, the formation of persulfides could also impact sodium homeostasis. The lipid phosphatase PTEN is inhibited when incubated with polysulfides (Greiner et al., 2013). It was observed that the inhibited form of the enzyme has an internal disulfide, possibly formed with the intermediacy of a persulfide in the protein, which is attacked by a neighboring cysteine to release H_2_S/HS^−^.

Last, the formation of a persulfide in certain catalytic cysteines allows the addition of sulfur atoms to several molecules of utmost anabolic or catabolic interest. As mentioned earlier, the external sulfur of the persulfides in specialized cysteine desulfurases is able to be transferred to fulfill the synthesis of several cofactors (Mueller, 2006). For instance, the synthesis of iron-sulfur clusters requires a desulfurase and is assisted by a particular multiprotein machinery (Lill and Freibert, 2020; Bandyopadhyay and Outten, 2022). A scaffold protein accepts the sulfur of the persulfide formed in the desulfurase, the iron atoms and electrons from reductants in a coordinated way to form the nascent [Fe_2_S_2_] clusters. In H_2_S/HS^−^ and cysteine catabolic pathways, under less strict conditions, the formation of persulfides in thiosulfate sulfurtransferases and MPSTs enables the transfer of sulfur atoms between acceptors to eliminate toxic forms of sulfur (Selles et al., 2019).

### Persulfides as potential protective modifications against irreversible thiol oxidation

Protein cysteines are susceptible to several posttranslational modifications, including sulfenylation (RSOH), sulfinylation (RSO_2_H), sulfonylation (RSO_3_H), thiolation or disulfide formation (RSSR, e.g., glutathionylation, RSSG), nitrosation (RSNO), prenylation, acylation and, as further explained herein, persulfidation (RSSH). These modifications can potentially change the properties of the original cysteine residue and lead to alterations in the functionality of the protein. Several of these modifications, including glutathionylation and persulfidation, can be reverted back to the thiol state by suitable reductants such as thioredoxin or glutaredoxin (Dóka et al., 2016; Wedmann et al., 2016).

The formation of RSO_2_H (with the exception of sulfiredoxin substrates) and RSO_3_H oxidation products is considered irreversible. In contrast, the oxidation of persulfides forms the products RSSO_2_H and RSSO_3_H, that can be reduced back to thiols with suitable reductants, thus protecting the original thiol from irreversible oxidation (Greiner et al., 2013). In addition, persulfides are excellent one-electron reductants (Filipovic et al., 2018). They react with one-electron oxidants forming relatively stable RSS^•^ radicals, which decay mainly by dimerization to the tetrasulfide RSSSSR (Everett et al., 1994).

### Methods for persulfide detection

Studying the biochemistry of persulfides demands specific and sensitive techniques for detection and quantitation. Many methods used for the detection of persulfides exploit their nucleophilicity. For isolated LMW components or proteins, the persulfides can be alkylated with electrophilic reagents such as iodoacetamide, monobromobimane or N-ethylmaleimide. This modification slows the decay of the persulfide, and allows mass spectrometric analysis that will indicate the extra sulfur. The mixed disulfide can then be reduced with dithiothreitol or tris(2-carboxyethyl)phosphine to the thiol, and the change in mass will be noticeable (Cuevasanta et al., 2015, 2019). Importantly, caution should be exerted regarding the stability of the disulfide products, and the electrophilic reagent should be chosen with care (Bogdándi et al., 2019; Schilling et al., 2022). Similar approaches based on persulfide nucleophilicity are used for in-gel detection of protein persulfides (Sen et al., 2012; Gao et al., 2015). Variations of these strategies can be applied to more complex cellular samples. Proteomic methods react the sample with a iodoacetyl-biotinylated reagent, isolate the biotinylated proteins with immobilized streptavidin, elute either proteins or tryptic peptides with a reductant or by UV-photorelease, and identify the peptides by LC-MS/MS (Dóka et al., 2016; Longen et al., 2016; Fu et al., 2020).

**FIGURE 3:**
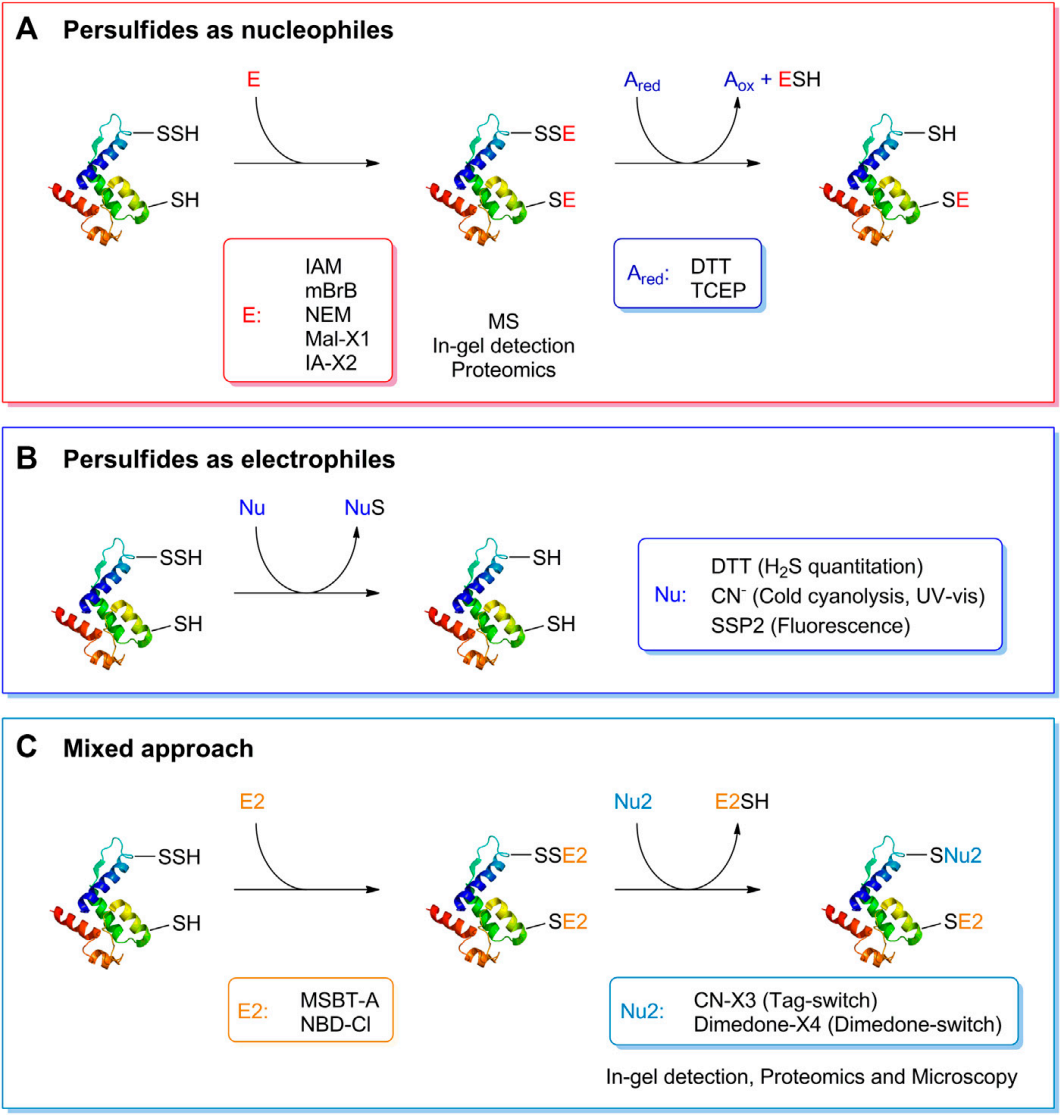
Detection of persulfides based on their reactivity. **(A)** The nucleophilicity of persulfides is exploited by means of the reaction with electrophilic alkylating agents such as iodoacetamide (IAM), monobromobimane (mBrB), N-ethylmaleimide and functionalized derivatives of maleimide and iodoacetamide (Mal-X1 and IA-X2) to generate a mixed disulfide. On the other hand, thiols generate a thioether. A subsequent reduction with DTT or TCEP generates the thiol from the mixed disulfide. This strategy has been used to study the formation of persulfides in LMW thiols and proteins, combined with mass spectrometry detection, in-gel detection or proteomics approaches. **(B)** The electrophilicity of persulfides is used when detecting persulfides by DTT reduction and H_2_S quantification, in the cold cyanolysis method detecting the ultimate formation of ferric thiocyanate, and with the fluorogenic probe SSP2. **(C)** A mixed approach has also been devised that first uses electrophilic reagents such as MSBT-A or NBD-Cl that generate an activated disulfide. In a second step, a nucleophile, either a functionalized cyanoacetate derivative or dimedone derivative, specifically reacts with this activated disulfide that then labels the site covalently. These methods have been named Tag-switch and dimedone-switch, respectively, and have been used in combination with in-gel detection, fluorescence microscopy and proteomics approaches. Further information is provided in the text and in (Cuevasanta et al., 2022).

Other methods take advantage of the electrophilicity of persulfides and use nucleophilic reagents that react with the sulfane sulfur. Persulfides can be indirectly quantified by methods that measure the H_2_S/HS^−^ released by the treatment of samples with DTT (Filipovic et al., 2018). The cold cyanolysis method is based on the reaction of cyanide with persulfides to yield thiocyanate (Eq. 9), that in the presence of ferric iron yields red ferric thiocyanate (Wood, 1987). Other sulfane-sulfur containing molecules also yield thiocyanate, so appropriate controls are required. The fluorogenic probe SSP2 reacts with the sulfane sulfur in persulfides and releases a benzodithiolone and a fluorophore (Chen et al., 2013). A two-step mixed approach has also been utilized, consisting of reacting the persulfide with an electrophilic reagent such as 2-(benzo[d]thiazol-2-yl sulfonyl)acetic acid (MSBT-A) or 4-chloro-7-nitrobenzofurazan (NBD-Cl), generating an activated disulfide, that then reacts with a nucleophile such as a cyanoacetate-derivative or a dimedone-derivative, resulting in the selective labeling of persulfides. The methods have been called either tag-switch or dimedone-switch, and have been applied to in-gel detection, proteomics and microscopy (Wedmann et al., 2016; Kouroussis et al., 2019; Zivanovic et al., 2019). A schematic representation of the different methods for persulfide detection is depicted in Figure 3.

Although the methodological advances have provided several insights into biological persulfidation, many challenges remain. For instance, the quantification of persulfides and their derivatives is troublesome and requires high amounts of persulfidated protein or peptides. Besides, the intrinsic instability of persulfides complicates the generation of reliable standards, and the stability of the persulfide alkylation products has been questioned. Most importantly, proteomic methods that identify persulfidated proteins usually lose track of the persulfidation. Ideally, the method should keep a chemical trace of the persulfide. A more detailed discussion on the methods used for persulfide detection is given in (Cuevasanta et al., 2022).

## Discussion

Efforts are being made to understand the role of persulfides in catalysis and (patho)physiology. Despite the technical difficulties found when analyzing persulfides *in vivo*, some correlations have been reported between persulfide metabolism and disease states. For example, mutations that affect the activity of mitochondrial persulfide dioxygenase (ETHE1, the enzyme in charge of GSSH degradation) are responsible for ethylmalonic encephalopathy, an inborn error of metabolism that leads to neurodevelopmental complications (Kabil and Banerjee, 2012). Also, persulfide levels have been related to aging, cancer development and chemoresistance, as well as cardiovascular and neurodegenerative diseases (Nishida et al., 2017; Zivanovic et al., 2019; Giovinazzo et al., 2021). Detailed mechanistic explanations are mostly lacking, and clues on the physicochemical consequences of persulfide formation in different thiols could assist in finding the missing links.

The electrophilicity of persulfides, absent in thiols and H_2_S/HS^−^, is at the basis of their ability to transfer the sulfane sulfur to acceptor molecules. This ability determines the important roles assigned to persulfides in the biosynthesis of sulfur-containing cofactors, such as iron-sulfur clusters. Transpersulfidation processes also participate in the detoxification of H_2_S/HS^−^, and several enzymes in the mitochondrial pathway for H_2_S/HS^−^ consumption catalyze transpersulfidation steps, such as SQOR and rhodanese. Importantly, the reaction rate of a typical LMW persulfide (i.e., GSSH), with a typical LMW thiol (i.e., GSH) is 1.1 M^−1^ s^−1^ at neutral pH (Benchoam et al., 2020). Hence, there is ample room for catalysis. In addition to lowering the energetic barrier for a particular reaction, a certain protein environment could tailor the reaction of a nucleophile with the outer versus the inner sulfur of a persulfide for a transpersulfidation process, while other surroundings for the persulfide could favor an attack on the inner sulfur to promote H_2_S/HS^−^ formation.

Altering the nucleophilic reactivity is another way in which the formation of a persulfide could impact on the properties of the parent thiol molecule. While nucleophilicity is higher in LMW persulfides in comparison to thiols due to both increased availability and alpha effect, the scenario emerging from the few studies of protein persulfides available is much more complex. Indeed, in the case of the peroxiredoxin *Mt*AhpE, the reactivity of the persulfidated protein was higher than that of the thiol protein in the case of a non-specific electrophile, but lower in the case of the specific hydroperoxide substrates (Cuevasanta et al., 2019).

The higher acidity of LMW persulfides versus thiols could lead one to think that, in the case of a protein, a negative charge would appear upon persulfidation. However, although p*K*_a_ values for protein persulfides have not been reported so far, it is likely that the protein environment is able to tune the acidity of the persulfide as well as the acidity of the thiol, leading us to sound a note of caution against generalizations.

Another way in which persulfide formation could impact the properties of the parent protein is through the bulk of the additional sulfur atom, as exemplified by aquaporin 8 (Bestetti et al., 2018).

Last, at least some of the biological effects of persulfidation could be due to the particular products formed from persulfides in comparison to the parent thiols. For example, persulfides would not lead to irreversible oxidation products (they can revert back to thiol with reductants), while several products formed by thiol oxidation are irreversible. This could be one of the mechanisms explaining the protective effects of persulfidation.

The reactivity of several persulfides of biological interest is being unveiled. The physicochemical characterization assists in rationalizing their possible roles, and results should be evaluated cautiously, considering particular persulfides. Hopefully, the next few years will consolidate the assessment of the biological consequences of persulfidation from solid kinetic and thermodynamic grounds.

## Footnotes

1 Hydropersulfide (RSSH) is the protonated species. Its IUPAC names are hydridodisulfide, disulfanyl, or dithiohydroperoxide. Persulfide anion is the deprotonated species (RSS^−^). Herein, the term “persulfide” is used for the mixture of RSSH and RSS^−^ in aqueous solution.
